# ﻿*Metapetrocosmeanavicularis*, a new species of Gesneriaceae in Vietnam

**DOI:** 10.3897/phytokeys.261.160454

**Published:** 2025-08-07

**Authors:** Qi-Yang Li, Ke Tan, Truong Van Do, Zi-Bing Xin, Xin-Xiang Bai, Song-Tao He, Fang Wen

**Affiliations:** 1 College of Forestry, Guizhou University, CN-5500252, Guiyang, Guizhou, China Guangxi Institute of Botany, Guangxi Zhuang Autonomous Region and Chinese Academy of Sciences Guilin China; 2 Guangxi Key Laboratory of Plant Conservation and Restoration Ecology in Karst Terrain, Guangxi Institute of Botany, Guangxi Zhuang Autonomous Region and Chinese Academy of Sciences, CN-541006, Guilin, Guangxi Zhuang Autonomous Region, China Guizhou University Guiyang China; 3 National Gesneriaceae Germplasm Resources Bank of GXIB, Gesneriad Committee of China Wild Plant Conservation Association, Gesneriad Conservation Center of China (GCCC), Guangxi Institute of Botany, Botanical Garden, Guangxi Zhuang Autonomous Region and Chinese Academy of Sciences, CN-541006, Guilin, Guangxi Zhuang Autonomous Region, China Botanical Garden, Guangxi Zhuang Autonomous Region and Chinese Academy of Sciences Guilin China; 4 Vietnam National Museum of Nature, Vietnam Academy of Science and Technology, 18th Hoang Quoc Viet, Hanoi, Vietnam Vietnam National Museum of Nature, Vietnam Academy of Science and Technology Hanoi Vietnam; 5 Graduate University of Science and Technology, Vietnam Academy of Science and Technology, 18th Hoang Quoc Viet Road, Cau Giay, Hanoi, Vietnam Graduate University of Science and Technology Hanoi Vietnam

**Keywords:** Didymocarpoideae, Flora of Vietnam, *Metapetrocosmeatamiana*, morphology, phylogeny

## Abstract

Based on morphological comparisons, literature reviews, and molecular systematic studies, a new species from South Vietnam, *Metapetrocosmeanavicularis* F.Wen, T.V.Do, Z.B.Xin & K.Tan, has been confirmed. Morphologically, this new species resembles *Metapetrocosmeatamiana* (B.L.Burtt) Yin Z.Wang & P.W.Li in leaf peltate, bilabiate corollas, arcuate filaments, and slightly curved capsule, while it is distinguished from *M.tamiana* in having leaf blade ovate to ovate-elliptic, boat-shaped bracts 6–9 mm long, the ratio of corolla lobes length to corolla tube length consistently 1 : 3, corolla tube gradually narrowed from mouth to base, subcylindrical-infundibulate, 10–20-flowered or more, filaments pubescent near the top. Molecular phylogenetic analyses using the combined dataset of *trn*L-F and ITS sequences show that the new species is also most closely related to *M.tamiana*. The conservation status of this new species is assessed as Least Concern (LC).

## ﻿Introduction

Vietnam, characterized by its unique geographical location, complex and elongated topography, and diverse climatic conditions, serves as a critical distribution area for the diversity of Gesneriaceae plants (Myers et al. 2000; Chen et al. 2020; Tan et al. 2020), with particular emphasis on *Metapetrocosmea* W.T.Wang. The genus consists of 12 species, four of which are distributed in China, whereas the remaining eight are endemic to Vietnam (Zheng et al. 2023; GRC 2025; Nguyen et al. 2025; Tan et al. 2025; Wen et al. 2025). *Metapetrocosmea* initially described in 1981 as a monotypic genus endemic to Hainan Island, China, was distinguished from *Petrocosmea* Oliv. based on its slightly elongated rhizome, subfiliform and longer filaments, villous anthers, divaricate thecae and subspheroidal capsules (Chen 1935; Wang 1981).

*Metapetrocosmea* underwent multiple systematic revisions, which have clarified its classification framework. Based on morphological, cytological, and phylogenetic analyses, Möller et al. (2016) reclassified five species, which had been previously transferred from *Chirita* Buch.-Ham. ex D.Don to *Primulina* Hance, into *Deinostigma* W.T.Wang & Z.Yu Li (1992). Phylogenetic analyses revealed that *Deinostigma* forms a strongly supported sister group relationship with the monotypic genus *Metapetrocosmea*. Shortly thereafter, Möller et al. (2020) provided additional morphological and molecular evidence to substantiate the distinctiveness of *Deinostigmacicatricosa* (W.T.Wang) D.J.Middleton & Mich.Möller and *D.minutihamata* (D.Wood) D.J.Middleton & H.J.Atkins (Wood 1972; Wang 1981; Weber et al. 2011). Subsequently, the continuous publication of new species has further expanded the circumscription of *Deinostigma* (Shui et al. 2020; Le et al. 2022). In 2022, based on molecular and morphological evidence, and in accordance with the International Code of Botanical Nomenclature’s priority rule, *Deinostigma* was merged into *Metapetrocosmea* (Li et al. 2022, 2024).

In 2018, during joint fieldwork in Dak Rong Natural Reserve, Vietnam, a species resembling a *Metapetrocosmea* species was discovered. After checking relevant papers (Phuong 2017; Shui et al. 2020; Le et al. 2022; Li et al. 2022), collecting samples of various herbaceous plants (e.g., from E, P, IBK, IBSC, KUN, NMNH and PE), and conducting phylogenetic analyses, it is described as a new species here.

## ﻿Materials and method

### ﻿Morphological comparisons

Detailed anatomical photographs and voucher specimens of this species were obtained through field documentation, involving systematic observation and documentation of its morphological characteristics. Subsequently, the morphological descriptions of related taxa were refined by integrating data from seminal literature and recent studies (Burtt 1999; Hickey and King 2000; Harris and Harris 2001; Phuong 2017; Li et al. 2022). Morphological characters of the species were compared with the digitized type specimens of related species deposited in herbaria E (https://www.rbge.org.uk/), NMNH (http://www.nmnh.si.edu/), and the images of similar species on PPBC (https://ppbc.iplant.cn/), as well as with morphological descriptions in the primary literature.

### ﻿DNA extraction, PCR, and sequencing

Traditional taxonomy is unable to accurately delimit the relationship between this new species and its close relatives. To address this, we will integrate molecular biological approaches. By obtaining the gene sequences of this new species and its close relatives, we aim to further investigate and enhance the accuracy of their phylogenetic relationships. Species’ leaf samples collected from the place of origin were quickly dried with silica gel for DNA extraction (Chase and Hills 1991). The nuclear ribosomal internal transcribed spacer (ITS) and chloroplast DNA sequences (*trn*L-F) of these samples were amplified by polymerase chain reaction (PCR), using the primers in the research of Taberlet et al. (1991) and White et al. (1990).

### ﻿Phylogenetic analysis

Based on previous research, the *trn*L-F and ITS sequences for nine *Metapetrocosmea* species were downloaded from Genbank. Two *Paraboea* species and two *Agalmyla* species were treated as outgroups (Table 1). All sequences were aligned using MAFFT v.7.5.1.1 (https://mafft.cbrc.jp/alignment/server/) (Katoh and Standley 2013), and conserved regions were selected using Gblock. The substitution saturation index (Iss) of the data matrix was evaluated using DAMBE v5.3.19 (Xia 2013), yielding Iss < Iss.c and P = 0.0000 (< 0.05), indicating no saturation; therefore, the dataset is suitable for phylogenetic tree construction. Multi-gene syndication was performed using Model Finder software, and polygenes were performed in PAUP*4.0 b10 (Swofford 2003). Subsequently, maximum likelihood (ML) analyses and BI inference in Phylosuite v.1.2.3 (Kalyaanamoorthy et al. 2017; Zhang et al. 2020; Xiang et al. 2023). The ML method employed 1,000 bootstrap replicates to assess the reliability of each node in the phylogenetic tree. It is generally accepted that if a node has a BS value greater than or equal to 70, then the branch is reliable (Guindon et al. 2010; Minh et al. 2013).The Bayesian inference (BI) method calculates posterior probabilities by applying optimal substitution models to each partition and independently estimating their parameters. Starting with random trees, it runs for 100,000,000 generations, sampling one tree every 10,000 generations (Zhang et al. 2020; Xiang et al. 2023). The first 25% of the trees are discarded as burn-in, and the remaining trees are used to generate consensus trees and calculate Bayesian posterior probabilities. Finally, we use the iTOL V4 version of the online tool (https://itol.embl.de) beautify the phylogenetic tree. The number of conserved sites, variable sites, and parsimony-informative sites for the combined dataset was obtained via MEGA 11.0.13.

**Table 1.** The voucher and GenBank accession numbers used in this study.

Species	Voucher	ITS	*trn*L-F
*Metapetrocosmeacicatricosa*	M.Möller & Y.G.Wei MMO 07-1148 [KN173] (E, IBK)	KU990890	KU990886
*Metapetrocosmeacycnostyla*	LPW2021004 (PE)	MZ265312	MZ325308
*Metapetrocosmeacyrtocarpa*	M.Möller & Y.G.Wei MMO 06-908 (E, IBK)	KU990889	KU990885
*Metapetrocosmeaeberhardtii*	M. Q. Han HMQ215182 (PE)	MZ265313	MZ325310
*Metapetrocosmeafasciculata*	M. Q. Han HMQ215181 (PE)	MZ265314	MZ325309
*Metapetrocosmeaminutihamata*	Xu, W.-B. s.n. [XWB] (IBK)	JX506925	JX506817
*Metapetrocosmeapeltata*	Y.G.Wei 07-702 (IBK)	HQ632968	HQ632872
*Metapetrocosmeapoilanei*	M. Poilane 3846 (PE)	MN627950	MN637601
*Metapetrocosmeatamiana*	Soviet-Vietnam Expedition (Liberec B.G., Czech Republic & St. Petersburg B.G) 01/114 [Cult. RBGE 19973431/19981743] (E)	KU990891	KU990887
*Metapetrocosmeanavicularis*	Q.Y.Li & K.Tan 241110 (IBK)	PV889409	PV878142
*Agalmylachalmersii*	Chapman sn [RBGE acc no. 19661971] (E)	MN843192	MN842997
*Agalmylaglabra*	RBGE-PNHE 1999 28 (E)	HQ632989	HQ632892
*Paraboeacrassifolia*	Möller & Wei MMO06-804a (E)	KU203841	KU203936
*Paraboeasinensis*	Möller MMO 06-949b (E)	JN934773	JN934731

## ﻿Results

### ﻿Morphological comparisons

**Figure 1.**
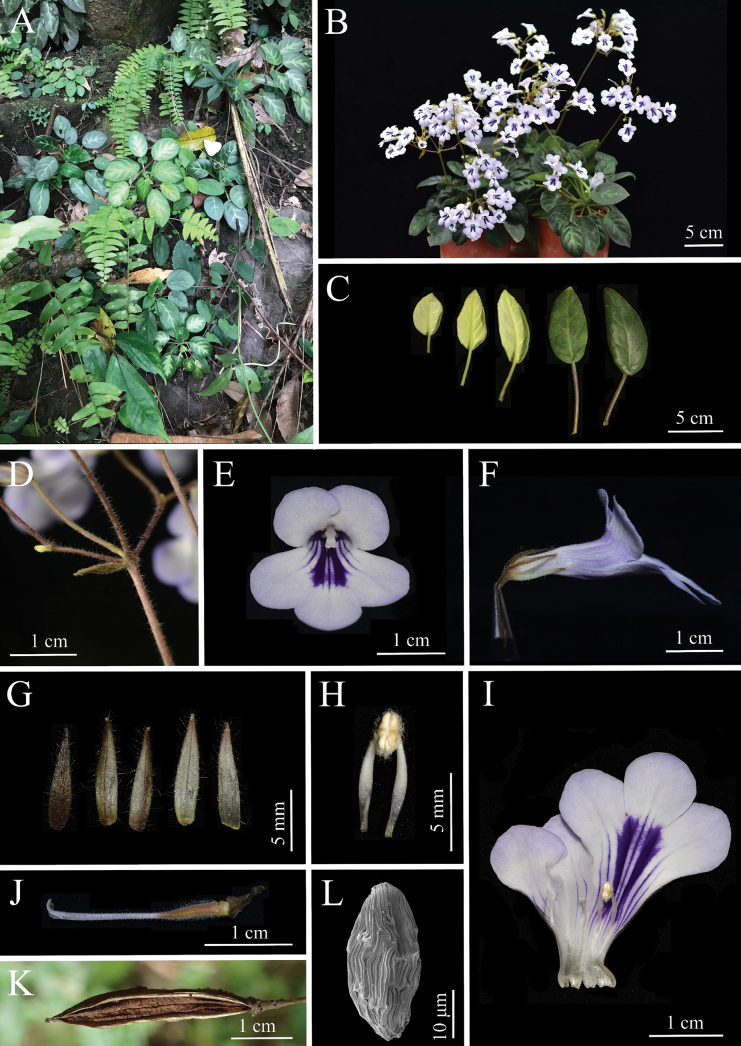
*Metapetrocosmeanavicularis*. A. Habitat; B. Plants; C. Leaves; D. Bracts; E. Frontal view of flower; F. Lateral view of corolla; G. Calyx lobes; H. Stamens; I. Opened corolla showing the interior surface of corolla tube, stamens and staminodes; J. Pistil and disc; K. Fruits; L. Micro-morphological characters of species seeds (Photos A, K were taken by Fang Wen; C, D by Qi-Yang Li and the rest were taken by De-Chang Meng).

**Figure 2.**
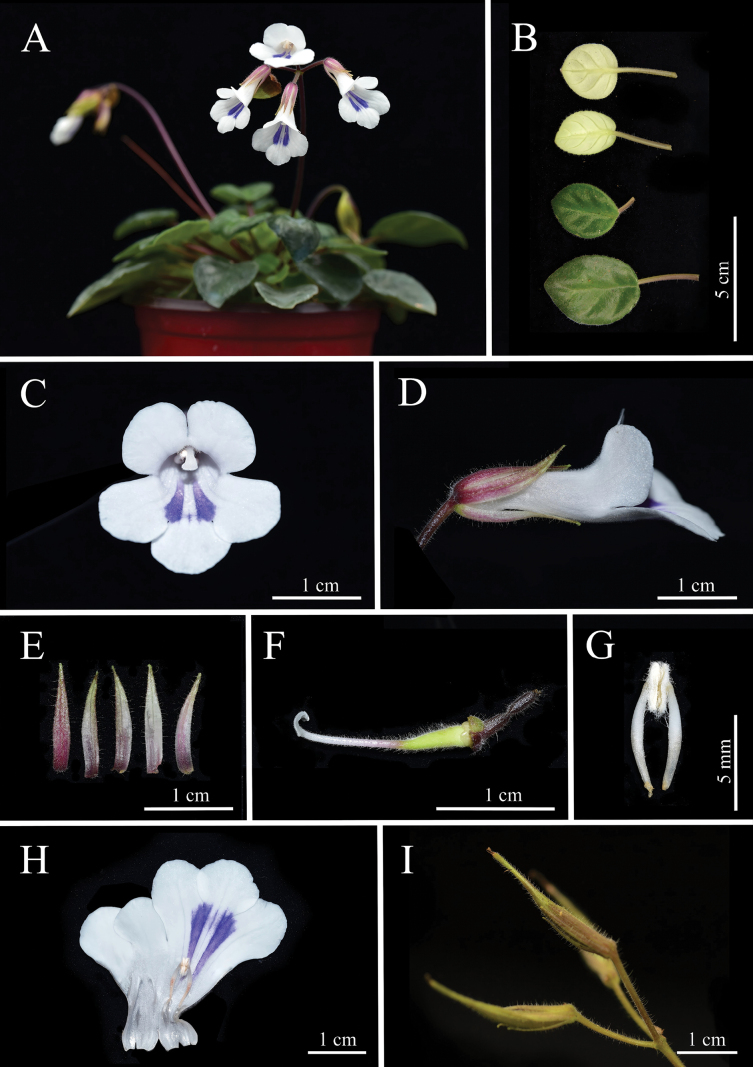
*Metapetrocosmeatamiana*. A. Plants; B. Leaves; C. Frontal view of flower; D. Lateral view of corolla; E. Calyx lobes; F. Pistil and disc; G. Stamens; H. Opened corolla showing the interior surface of corolla tube, stamens and staminodes; I. Fruits (Photos A–H were taken by De-Chang Meng).

The species of *Metapetrocosmea* (Subfam. Didymocarpoideae, Gesneriaceae) are mostly characterized by being perennial herbs, having alternate leaves, arcuate filaments, bilobed stigma and so on (Li et al. 2022). The new species, sharing traits, is morphologically similar to *M.tamiana*. However, it differs by having a larger plant and leaf size, boat-shaped bracts, more flowers, shorter calyx lobes and subcylindrical-infundibulate corolla tube, among others (Table 2, Figs 1, 2).

**Table 2.** Main morphological comparison between *Metapetrocosmeanavicularis* and *M.tamiana*.

Characters	*M.navicularis*	*M.tamiana*
Leaf blade shape	ovate to ovate-elliptic	cordate to nearly round
Leaf indumentum	densely puberulent adaxial	with mixture of long and short pubescence adaxial
Bracts length	6–9 mm	ca. 2 cm
Sepal lobes length (sepal lobes : corolla tube)	1 : 3	1 : 1
The number of flowers	10–20-flowered or more	4–6-flowered
Corolla tube	subcylindrical-infundibulate	distinctly infundibulate
Filaments indumentum	villous near the top	glabrous
Style indumentum	puberulent	pubescent
Style length : Ovary length	2 : 1	1 : 1
Stigma shape	arcuate	nutant

### ﻿Phylogenetic analysis

**Figure 3.**
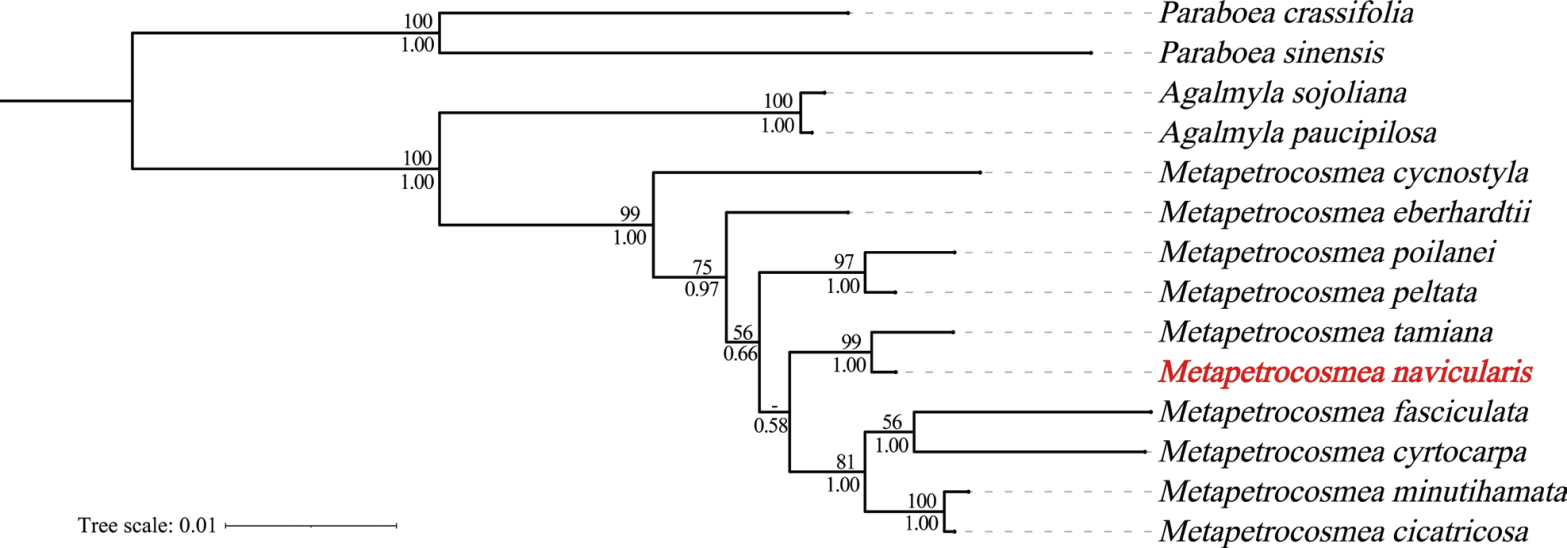
Phylogeny of the *Metapetrocosmea*. The majority consensus tree of the Bayesian Inference method based on ITS and *trn*L-F regions. The bootstrap values (BS) of ML and posterior probabilities (PP) of BI are listed at each node. Asterisks indicate maximum support and hyphens represent no support. The new species is highlighted in bold.

The combined dataset has 1,096 characters (783 *trn*L-F and 313 ITS), with 928 (85%) conserved sites, 168 (15%) variable sites, and 96 (8.8%) parsimony-informative sites. The best substitution models for *trn*L-F and ITS partitions, obtained via PhyloSuite v1.2.3, are GTR + G and GTR + I. Both ML and BI trees show the new species is most closely related to *Metapetrocosmeatamiana* (BS = 99%, PP = 1.00), with identical topologies (Fig. 3). This consistent topology across both analytical methods underscores the robustness of the phylogenetic placement, reinforcing the distinct evolutionary lineage of the new taxon within the genus.

### ﻿Taxonomic treatment

#### ﻿ Metapetrocosmea navicularis

Taxon classificationPlantaeLamialesGesneriaceae

F.Wen, T.V.Do, Z.B.Xin & K.Tan sp. nov.

FC3341FB-6E9A-5D9C-BB7E-5D0D2A467505

urn:lsid:ipni.org:names:77366813-1

Fig. 1

##### Type.

China • Guangxi: cultivated in Guilin Botanical Garden, introduced from Vietnam, Quang Tri Prov, Dak Rong Natural Reserve, on rock walls of a local waterfall, 15°40'41"N, 107°42'22"E, 190 m a.s.l., 10 Nov. 2024, *Q.Y.Li & K.Tan 241110* (holotype: IBK! [IBK00470816!]; isotypes: IBK! [IBK00470817!], VNMN!).

##### Diagnosis.

*Metapetrocosmeanavicularis* is similar to *M.tamiana* in macroscopic morphology, and phylogenetic analysis indicates that they are the closest relatives (Fig. 1). But it is easy to distinguish the new species from *M.tamiana* by its leaf blade ovate to ovate-elliptic (*vs.* cordate to nearly round, the following comparisons follow the same order), leaf densely puberulent adaxial (*vs.* with mixture of long and short pubescence adaxial), bracts 6–9 mm long (*vs.* ca. 2 cm long), the ratio of corolla lobes length to corolla tube length consistently 1 : 3 (*vs.* approximately 1 : 1), 10–20-flowered or more (*vs.* 4–6-flowered), corolla tube gradually narrowed from mouth to base, subcylindrical-infundibulate (*vs.* wider in upper part, abruptly contracted towards base, distinctly infundibulate), filaments pubescent near the top (*vs.* glabrous), style puberulent (*vs.* pubescent), and stigma arcuate (*vs.* nutant) (Table 2).

##### Description.

Perennial herb. Rhizome subcylindrical, ca. 4 cm long, 6–8 mm in diameter, perennial-growing rhizomes exhibit distinct abscission scars following leaf abscission. Leaves 8–12 or more, petiolate; petioles 3–6 cm long, ca. 3 mm in diameter, pale green, puberulous; leaf blade alternate, ovate to ovate-elliptic, 6–8 × 2.5–4 cm, dark green to green adaxially with pale green veins commonly, pale green abaxially, apex acute, base obtuse, peltate, margin crenate, densely puberulent; venation penninerved, lateral veins 3–5 pairs, inconspicuously impressed adaxial veins, prominently raised abaxial. Inflorescence cymes, axillary, 10–20-flowered or more. Peduncles densely pubescent, 6–12 cm long, ca. 4 mm in diameter; pedicels 1–3 cm long, ca. 2 mm in diameter, densely pubescent. Bracts 2, opposite, distinctly navicula, namely generally oblong-elliptic, base broader, apex attenuate being acuminate, resembling a small boat, 5–9 × 2–3 mm, margin entire but slightly reflexed, densely pubescent, deciduous, generally not persistent; calyx 5-parted to the base, lobes nearly equal in length, ca. 8 × 2 mm, lanceolate, dark green, apex acute, outside villosulous, inside glabrous, margin ciliate. Corolla pale purple to white, ca. 3.5 × 2.5 cm, bilabiate; upper lip 2-lobed, split to the midpoint of the corolla tube, lobes ca. 9 × 6 mm; lower lip 3-lobed, central lobe more rounded than lateral lobes, lobes ca. 9 × 8 mm; corolla tube gradually narrowed from mouth to base, subcylindrical-infundibulate, with two dark purplish-blue lines that stretch from either side of the median lobe of the corolla down into the throat. Stamens 2, ca. 7 mm long, inserted at 8 mm from the base of the corolla; filaments linear, white, villous near the top, arcuate; anthers oblong, pale yellow, ca. 3 mm long, lanate; staminodes 2, ca. 4 mm long, ca. 1 mm in diamete, inserted at 8 mm from the base of the corolla, villous near the top, slightly curved. Disc annular, pale yellowish-green, ca. 1 mm high, glabrous. Pistil ca. 1.8 cm long; ovary linear, ca. 6 mm long, densely pubescent; style linear, ca. 1 cm long, white, densely puberulent; stigma obtrapeziform, emarginate. Capsule obliquely narrowly oblong, ca. 3 cm long, dehisces along the ventral suture; seed surface, longitudinal prominent ribs are further linked to become somewhat reticulate.

##### Phenology.

The peak flowering period occurs in December, with flowering from November to January and fruiting from February to March in the nursery of the botanical garden. Whether the natural flowering and fruiting periods of wild populations differ from those of cultivated populations in botanical garden requires long-term field observations for confirmation.

##### Vernacular name.

“Hình dáng giống thuyền” (Understood as the bracts of this species are interpreted as naviculate shape). Its Chinese name is Chuán Bāo Dùn Yè Jù Tái (Chinese pronunciation); 船苞盾叶苣苔.

##### Distribution and habitat.

*Metapetrocosmeanavicularis* is relatively common in moist with abundant populations and shaded habitats under evergreen broad-leaved forests at an altitude of approximately 190 m in its type locality. This species grows in moist rock crevices, with accompanying species mainly including some ferns and other herbaceous plants.

##### Additional examined specimens.

*Metapetrocosmeatamiana*, Vietnam • Vinh Phu Province, Tam Dao, 4 Nov. 1997, Soviet-Vietnamese Expedition 1986, No. 114. 19973431*A (holotype: E!); Tam Dao, 1998, *Soviet-Vietnamese Expedition 1986, No. 114. 15738* (E!); Tam Dao, 13 Jan. 1999, *L.E. Skog & J.K. Boggan 7851* (US!). *Metapetrocosmeanavicularis*, Vietnam • Quang Tri Province, Dak Rong Natural Reserve, growing on rocky cliffs by the side of the trail on the hill slope, 15°40'41"N, 107°42'22"E, 190 m a.s.l., 19 Mar. 2018, *Van Truong Do et al. VMN-CN 1069* (VNMN!).

##### Conservation status.

The species inhabits the Dak Rong Nature Reserve, Vietnam, an area of high biodiversity. Thanks to the favorable growing environment, the species has developed abundant populations here, resulting in a thriving population size. This richness in numbers is further supported by effective conservation and management efforts in the region. Following the IUCN Red List Categories and Criteria (IUCN Standards and Petitions Committee 2024), our preliminary assessment result is Least Concern (LC).

##### Note.

It is very special because its bracts of this species are interpreted as naviculate shape, which is the first discovered in *Metapetrocosmea*. The character can also help distinguish it from all other species of *Metapetrocosmea* from Vietnam and China. From a macroscopic morphological perspective, this new species is indeed closest to *M.tamiana*, which is currently known to be distributed in Tam Dao National Park near Hanoi City in northern Vietnam. This represents a significant long-distance geographic isolation from the new species, which is distributed in southern Vietnam. We know that within subfam. Didymocarpoideae of Gesneriaceae, microenvironmental variations across different terrains and landforms can give rise to rich species diversity (Tan et al. 2020). Among Chinese taxonomists, there’s even a widely circulated saying about the important characteristic of Gesneriaceae species diversity: “one species per mountain”, “one species per gully”, “one species per cave”, a point particularly prominent in the closely related genus *Primulina* (Wei 2018; Wei et al. 2022, 2023). Considering the geographic isolation, there are also many readily discernible characteristics between this new species and *Metapetrocosmeatamiana* (Table 2). For example, the plant size and leaf size of the new species are 2 to 3 times larger than the latter and are very stable, maintaining this difference even under long-term common garden cultivation. After common garden cultivation, the new species can have more than 10 flowers per inflorescence, with the entire plant producing 60 or more flowers, densely clustered on the inflorescence (Fig. 1B). In contrast, *M.tamiana* consistently has fewer than 10 flowers per inflorescence (around 4-6) and typically around 20 flowers per plant, with the flowers on the inflorescence being more scattered (Fig. 2A). In wild populations of this new species, more than 90% of individuals exhibit clear or indistinct white fishbone-patterned leaf veins (Fig. 1A), while the latter has entirely green leaves without any white fishbone patterns (Fig. 1C). However, since these traits are quantitative or semi-qualitative, they are not ideal for definitively distinguishing the two species. In contrast, the new species’ naviculate bract shape and the stable ratio of corolla lobe length to corolla tube length consistently being 1 : 3, along with its pubescent filaments, are good qualitative characteristics for clearly differentiating the two species (Figs 1F, 2D). In terms of flower size, the flowers of this new species are significantly larger in diameter than those of *M.tamiana*: measurements show that the flower diameter of the new species is usually around 2.5 cm, while that of *M.tamiana* is generally only ca. 2 cm (Figs 1E, 2C). This size difference is clearly distinguishable to the naked eye. Turning to the blue-purple nectar guides on the inner surface of the corolla, although they appear similar, there are essential differences between the two species. The nectar guides of *M.tamiana* exhibit poor color stability: under high-temperature conditions in summer, their color fades gradually, eventually becoming extremely pale, almost white, and once this fading occurs, it is difficult to recover in a short period. In contrast, the nectar guides of the new species show extremely strong color stability; even in the same high-temperature environment, their blue-purple color remains vivid without fading or lightening. This characteristic also serves as an important diagnostic marker for distinguishing the two species (Figs 1I, 2H). Moreover, in conjunction with phylogenetic analysis, we acquired the sequences of the type locality of *M.navicularis* and formed a fully supported clade in the phylogenetic tree (Fig. 3).

## Supplementary Material

XML Treatment for Metapetrocosmea navicularis

## ﻿Additional information

### Use of AI

No use of AI was reported.

### Author ORCIDs

Qi-Yang Li https://orcid.org/0009-0006-9336-7718

Ke Tan https://orcid.org/0000-0002-9036-163X

Truong Van Do https://orcid.org/0000-0002-0585-5513

Zi-Bing Xin https://orcid.org/0000-0002-0062-6930

Xin-Xiang Bai https://orcid.org/0000-0003-2449-6664

Song-Tao He https://orcid.org/0009-0005-5223-7522

Fang Wen https://orcid.org/0000-0002-3889-8835

### Data availability

All of the data that support the findings of this study are available in the main text or Supplementary Information.
